# Highly specific amyloid and tau PET ligands for ATN classification in suspected Alzheimer’s disease patients

**DOI:** 10.1007/s12149-025-02018-7

**Published:** 2025-01-28

**Authors:** Hiroshi Matsuda, Haruo Hanyu, Chikako Kaneko, Masato Ogura, Tensho Yamao

**Affiliations:** 1 Drug Discovery and Cyclotron Research Center, Southern Tohoku Research Institute for Neuroscience, 7-61-2 Yatsuyamada, Koriyama, Fukushima 963-8052 Japan; 2 Department of Biofunctional Imaging, Fukushima Medical University, 1 Hikarigaoka, Fukushima City, Fukushima 960-1295 Japan; 3 Dementia Research Center, Tokyo General Hospital, 3-15-2 Egota, Nakano-Ku, Tokyo, 165-0022 Japan; 4 Department of Neurology, Southern Tohoku General Hospital, 7-115 Yatsuyamada, Koriyama, Fukushima 963-8052 Japan; 5 Department of Radiological Sciences, School of Health Science, Fukushima Medical University, 10-6 Sakae, Fukushima City, Fukushima 960-8516 Japan; 6 Southern Tohoku Research Institute for Neuroscience, Metropolitan Office, Shinotemachi-Building, 6F (621), 2-2-1, Otemachi, Chiyoda-ku, Tokyo, 100-0004 Japan

**Keywords:** Alzheimer’s disease, Amyloid imaging, Tau imaging, Positron emission tomography

## Abstract

**Objective:**

This study aims to accurately classify ATN profiles using highly specific amyloid and tau PET ligands and MRI in patients with cognitive impairment and suspected Alzheimer’s disease (AD). It also aims to explore the relationship between quantified amyloid and tau deposition and cognitive function.

**Methods:**

Twenty-seven patients (15 women and 12 men; age range: 64–81 years) were included in this study. Amyloid and tau PET scans were performed using ^18^F-NAV4694 and ^18^F-MK6240, respectively. For each patient, amyloid and tau PET images were visually assessed and classified as either amyloid-positive or amyloid-negative, and as ^18^F-MK6240 Braak stage 0 (tau-negative) or Braak stages I–VI (tau-positive). Voxel-based morphometry of three-dimensional T1-weighted MRI was used to evaluate neurodegeneration. Amyloid and tau depositions were quantified using the Centiloid scale and standardized uptake value ratio (SUVR), respectively. Global cognitive function was assessed with the Mini-Mental State Examination (MMSE).

**Results:**

Patients were categorized into seven ATN profiles. Six patients (22%) exhibited a normal AD biomarker profile, 15 patients (56%) fell within the Alzheimer’s continuum, and 14 patients (52%) were diagnosed with AD. Additionally, six patients (22%) displayed non-AD pathological changes. Positive and negative findings of amyloid and tau PET were concordant in 24 patients (89%). Among the 14 patients diagnosed with AD, the Centiloid scale for amyloid deposition did not show a significant negative correlation with MMSE scores (*r* = 0.269, *p* = 0.451). In contrast, the SUVR for tau deposition in the neocortex exhibited a significant negative correlation (*r* = −0.689, *p* = 0.014), while tau deposition in the mesial temporal region did not show a significant correlation (*r* = 0.158, *p* = 0.763).

**Conclusion:**

Highly specific amyloid and tau PET scans, along with MRI, can be utilized to accurately classify ATN profiles in patients with cognitive impairment and suspected AD. The discordance in amyloid and tau PET findings in three patients allowed for a more precise AD diagnosis. Furthermore, tau PET imaging provided insight into the propagation of tau deposition in the neocortex beyond the mesial temporal region, which is associated with cognitive decline.

## Introduction

With the introduction of insurance coverage for amyloid PET as a companion diagnostic tool in anti-amyloid-β antibody therapy for Alzheimer’s disease (AD) in Japan, accurately diagnosing AD in the mild cognitive impairment or mild dementia stages has become increasingly important. In our multicenter study [1], the diagnostic accuracy for these early stages of AD without the use of amyloid PET was only 52%. Amyloid positivity, determined through cerebrospinal fluid (CSF) testing or amyloid PET, is essential for determining eligibility for anti-amyloid-β antibody therapy.

In addition, neurofibrillary tau deposits—another pathognomonic feature of AD—can now be assessed through CSF testing or PET imaging. Jack et al. [2] proposed a research framework for a biological definition of AD that integrates these biomarkers, which may prove useful in guiding therapeutic strategies. This framework is referred to as the ATN classification, where “A” stands for amyloid, “T” for tau, and “N” for neurodegeneration. Neurodegeneration is assessed using magnetic resonance imaging (MRI) or fluorodeoxyglucose PET.

Compared to CSF testing, PET imaging can not only identify the sites of amyloid-βdeposition but also quantify the amount of this deposit, making it particularly valuable in determining whether to discontinue anti-amyloid-β antibody therapy [3]. In PET-based ATN classification for AD, the choice of amyloid and tau PET ligands is critical. Ligands that are highly specific for amyloid-β or those that bind specifically to 3R/4R tau, which is characteristic of AD, are expected to enhance the accuracy of ATN classification, especially in the early stages of AD [4].

In the present study, we aimed to achieve accurate ATN classification using ligands highly specific for amyloid-β and pathological tau, along with MRI, in patients with cognitive impairment and suspected AD. Additionally, we sought to quantify amyloid-β and tau deposition to evaluate the impact of these pathological protein deposits on cognitive function.

## Materials and methods

### Patient recruitment

In total, 29 Japanese patients (17 women and 12 men; age range: 64–81 years) were recruited from a specialized dementia section at Southern Tohoku General Hospital. General cognition was assessed using the Mini-Mental State Examination (MMSE) [5]. The inclusion criteria were as follows: cognitive impairment suspected to be due to AD based on the clinical criteria for AD established by the National Institute on Aging and the Alzheimer’s Association [6], prior to amyloid PET imaging; and a brain MRI scan (three-dimensional T1-weighted, T2-weighted, and FLAIR imaging) performed within 90 days before registration. The exclusion criteria included the absence of cognitive decline and gross brain lesions, such as brain tumors, cerebrovascular malformations, or cortical infarctions, as identified on MRI.

One patient who passed the screening withdrew consent before undergoing the PET scan. Another patient canceled participation due to scheduling conflicts. Ultimately, 27 patients (15 women and 12 men; age range: 64–81 years) were included in this study.

### PET/CT imaging

For amyloid PET, ^18^F-NAV4694 was injected intravenously as a slow bolus into an antecubital vein at a mean ± standard deviation (SD) dose of 300 ± 13 MBq (range: 259–321). For tau PET, ^18^F-MK6240 was injected at a mean ± SD dose of 191 ± 9 MBq (range: 179–203). The interval between the two PET scans was 23 ± 11 days (range: 7–49). A 20-min list-mode PET scan began 50 min after the injection of ^18^F-NAV4694, and a 30-min list-mode PET scan began 90 min after the injection of ^18^F-MK6240, using a hybrid PET/CT scanner (ClariTom uMI 780; United Imaging Healthcare, Shanghai, China).

Image reconstruction was performed using a three-dimensional ordered subsets expectation maximization algorithm with the following parameters: image matrix, 128; field of view, 256 mm; subsets, 20; iterations, 3; post-filter (Gaussian), 3-mm FWHM; attenuation correction, CT-based. The resulting voxel size was 2.0 × 2.0 × 3.0 mm^3^.

### MRI

MRI was performed for all patients using a 1.5-T scanner (Signa Explorer; GE Medical Systems, Chicago, USA) equipped with an HNS head coil. A volumetric turbo field echo T1-weighted structural sequence was acquired with the following parameters: number of sagittal slices, 102; TR, 10.1 ms; TE, 4.2 ms; inversion time, 400 ms; field of view, 256 × 256 mm; voxel size, 1.0 × 1.0 × 1.6 mm^3^; flip angle, 15°.

### Visual interpretation of PET and MRI

Amyloid PET images were visually assessed and dichotomously rated as either amyloid-positive or amyloid-negative by a board-certified nuclear medicine physician (HM). Tau PET images were visually assessed by the same physician using the ^18^F-MK6240 Braak staging system (0 = tau-negative, I–VI = tau-positive), according to previous investigations [7, 8] based on neuropathological neurofibrillary tangle staging [9, 10]. Voxel-based morphometry of three-dimensional T1-weighted MRI was used to evaluate neurodegeneration with the Voxel-Based Specific Regional Analysis System for Alzheimer’s Disease (VSRAD) software [11]. Neurodegeneration was defined as a *Z*-score color map greater than 2 in the mesial temporal region and/or temporoparietal cortex compared to a normal database.

### Quantitative analysis of PET

For amyloid PET, the Centiloid scale [12] was calculated using Amyquant^®^ software [13], which also detects significant local amyloid deposition compared to an amyloid-negative control database.

For tau PET, the standardized uptake value ratio (SUVR) of regional tau deposition was estimated, with the cerebellar cortex as the reference region. Three regional tau masks, as reported by Villemagne et al. [14], were used:

1. Mesial temporal: Includes the entorhinal cortex, hippocampus, parahippocampus, and amygdala.
2. Temporoparietal: Includes the inferior and middle temporal gyri, fusiform gyrus, supramarginal and angular gyri, orbitofrontal cortex, gyrus rectus, posterior cingulate/precuneus, superior and inferior parietal lobules, and lateral occipital cortex.
3. Rest of the neocortex: Includes the dorsolateral and ventrolateral prefrontal cortex, superior temporal gyrus, and anterior cingulate cortex.

**Fig. 1:**
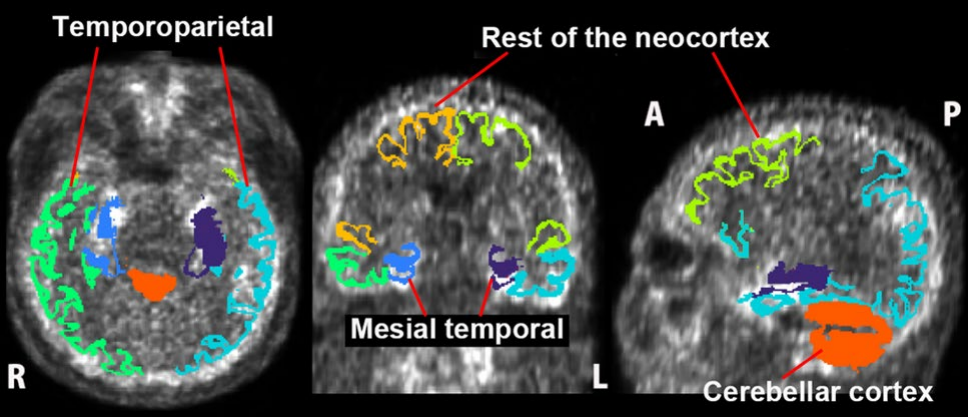
Masks for quantification of tau deposition. Three regional masks—mesial temporal, temporoparietal, and the rest of the neocortex—as well as a reference mask for the cerebellar cortex, were derived from 3 dimensional T1-weighted MRI. These masks were coregistered to tau PET scans using MRI-based native space FreeSurfer parcellations

A global tau deposition measure was calculated as the average SUVR across all three regions. Neocortical tau deposition was calculated as the average SUVR for the temporoparietal and the rest of neocortex regions. Braak staging progresses sequentially from the mesial temporal region to the temporoparietal region and then to the rest of the neocortex. The regional masks were generated from three-dimensional T1-weighted MRI images coregistered to tau PET using FreeSurfer parcellations in MRI-based native space [15] (Fig. 1).

### Statistical analysis

The relationships between MMSE scores and the amounts of amyloid or tau deposition were assessed using Pearson correlation coefficients, controlling for age and sex. All statistical analyses were performed using JMP version 18.1.1 (SAS Institute). If the *p* value is less than 0.05, it is judged as significant.

## Results

**Fig. 2:**
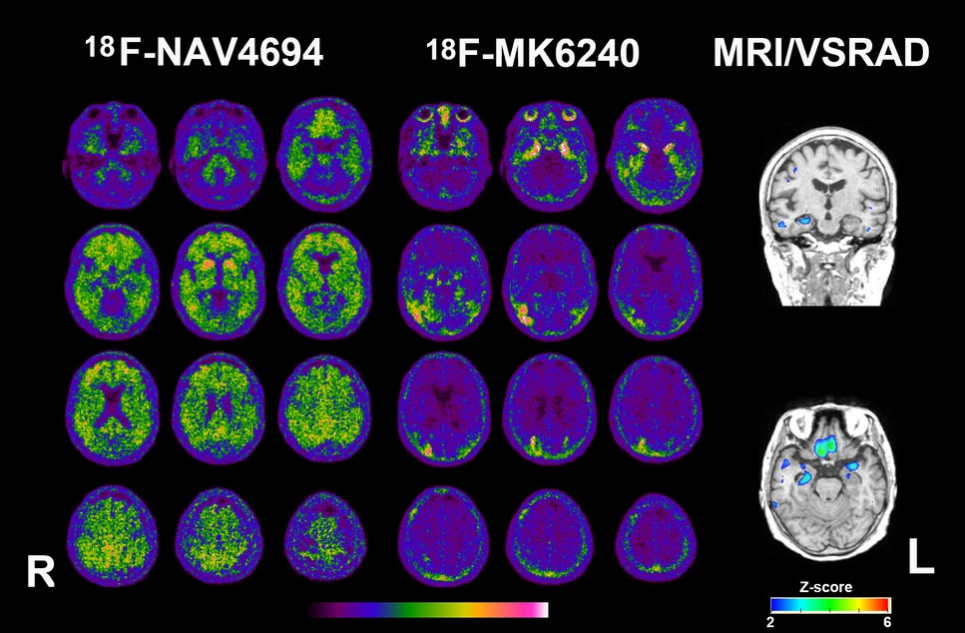
PET and MRI of a woman in her late 70s with AD (A+T+N+). Amyloid PET Centiloid scale is 86. Tau PET Braak stage is determined to be V, as tau deposition is observed in the bilateral medial temporal regions and the right temporoparietal cortex. The *Z*-score color map, analyzed using VSRAD software, demonstrates significant atrophy in the mesial temporal regions with a *Z*-score of 2 or higher

**Fig. 3:**
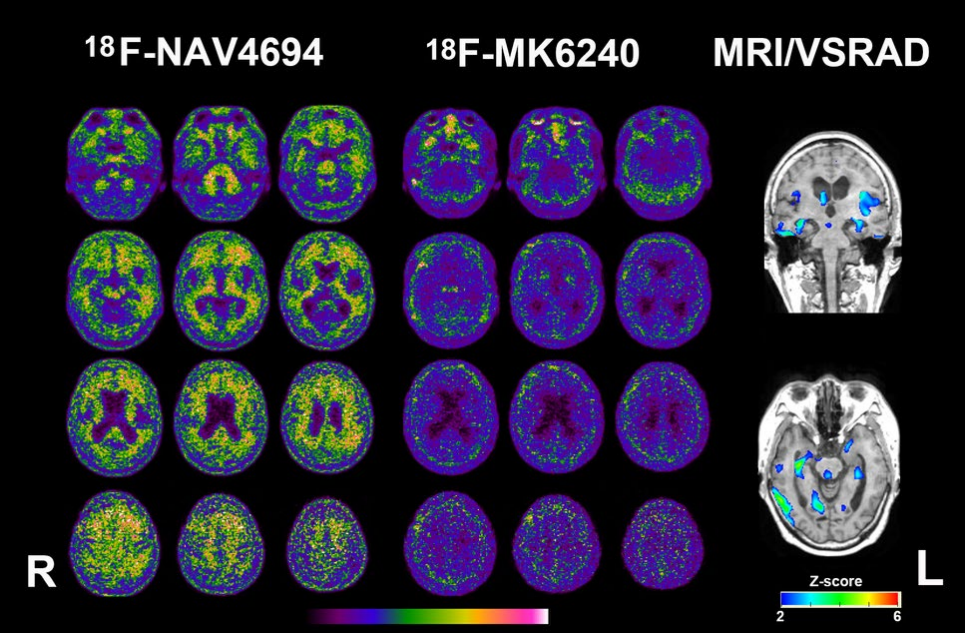
PET and MRI of a woman in her late 70s with Alzheimer’s and concomitant suspected non-Alzheimer’s pathologic change (A+T−N+). Amyloid PET Centiloid scale is 37. Tau PET is negative. The *Z*-score color map reveals significant atrophy in the mesial temporal regions with a *Z*-score of 2 or higher

**Fig. 4:**
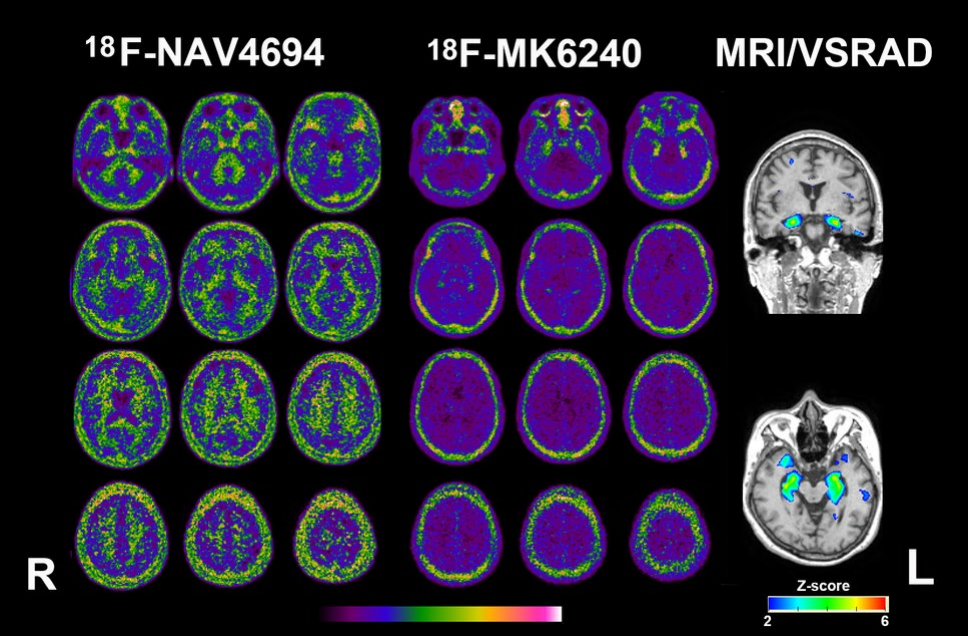
PET and MRI of a woman in her late 60s with primary age related tauopathy (A−T+N+). Amyloid PET is negative. Tau PET Braak stage is determined to be I, as tau deposition is observed only in the bilateral medial temporal regions. The *Z*-score color map, analyzed using VSRAD software, shows significant atrophy in the mesial temporal regions with a *Z*-score of 2 or higher

Adverse events were not observed following the administration of the PET ligands or immediately after the PET scans. The MMSE scores of the 27 patients were 25.2 ± 2.7, with a range from 17 to 30. Amyloid-positive findings were observed in 15 of 27 patients (56%), while tau-positive findings were observed in 16 of 27 patients (59%), with Braak stages distributed as follows: stage I in 1 patient, stage II in 3 patients, stage IV in 1 patient, stage V in 4 patients, and stage VI in 7 patients. In visual interpretation, the Centiloid scale for the amyloid-positive and amyloid-negative groups ranged from 37 to 114 (73 ± 24) and from −8 to 9 (− 2 ± 5), respectively, with no overlap between the two groups. Similarly, in visual interpretation, the global SUVR for the tau-positive and tau-negative groups ranged from 1.09 to 1.66 (1.25 ± 0.19) and from 0.84 to 1.08 (0.93 ± 0.08), respectively, also showing no overlap between the two groups. Concordance between amyloid and tau PET findings (both positive or both negative) was observed in 24 patients (89%). Significant atrophy in the mesial temporal region and/or temporoparietal cortex was observed in 18 patients (67%).The 27 patients were classified into seven ATN profiles as follows: A−T−N−, 6 patients; A+T+N−, 2 patients; A+T+N+, 12 patients; A+T−N+, 1 patient; A−T+N−, 1 patient; A−T−N+, 4 patients; A−T+N+, 1 patient (Table 1). Six patients (22%) displayed a normal AD biomarker profile, 15 patients (56%) fell within the Alzheimer’s continuum, 14 patients (52%) were diagnosed with AD, and 6 patients (22%) were characterized by non-AD pathological changes. Representative cases are shown in Figs. 2, 3, and 4.

**Table 1:** ATN classification results for studied patients

ATN profiles	Number of patients	Biomarker category	
A−T−N−	6	Normal AD biomarker	
A+T−N−	0	Alzheimer’s pathologic change	Alzheimer’s continuum
A+T+N−	2	Alzheimer’s disease	
A+T+N+	12		
A+T−N+	1	Alzheimer’s and concomitant suspected non Alzheimer’s pathologic change	
A−T+N−	1	Non-AD pathologic change	
A−T−N+	4		
A−T+N+	1

**Fig. 5:**
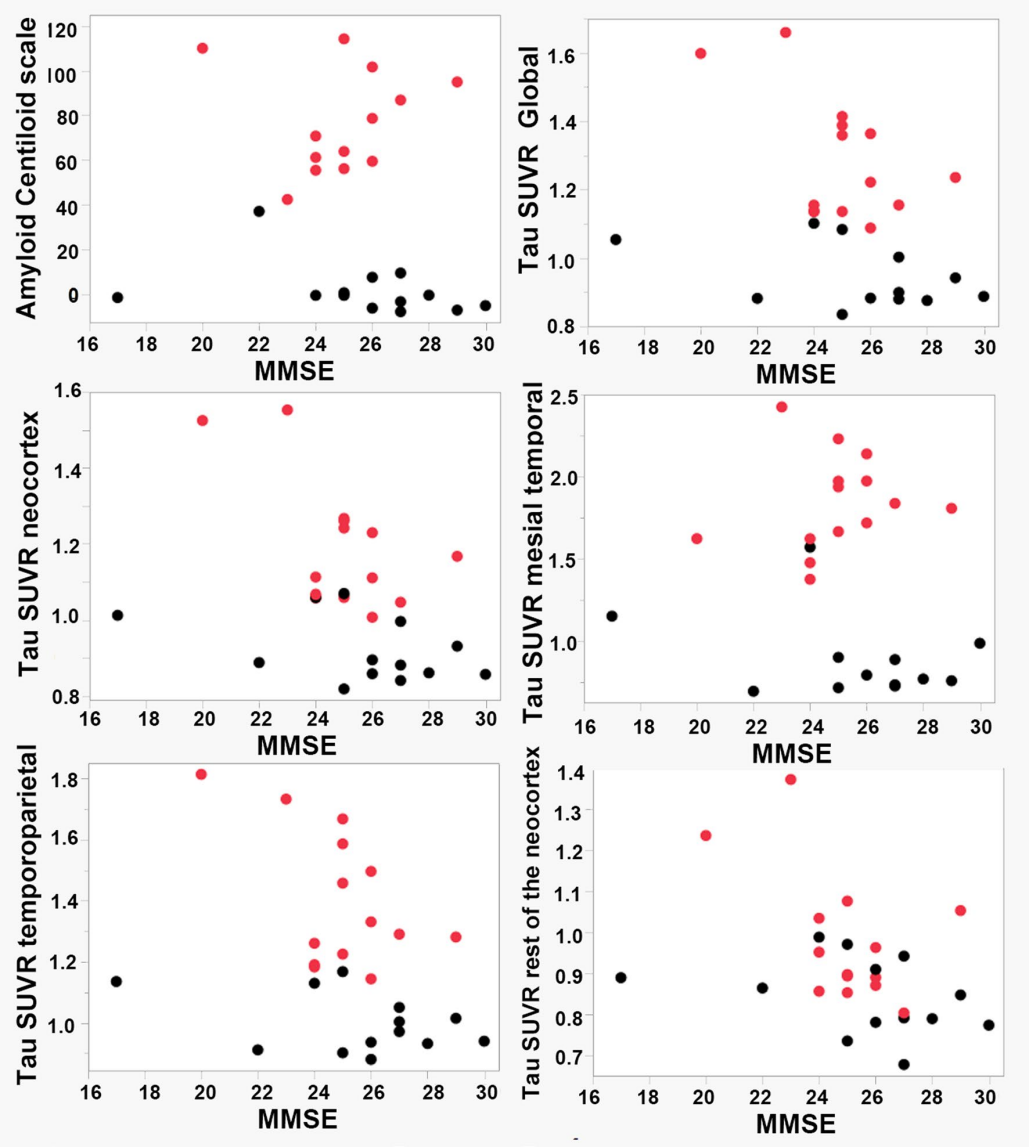
Scatterplot illustrating the association between MMSE scores and amyloid-PET Centiloid scale or tau-PET SUVRs. Tau-PET SUVR was calculated separately for the global, neocortical, mesial temporal, temporoparietal, and the rest of the neocortical regions. Red circles indicate cases diagnosed with Alzheimer’s disease (A+T+)

Across all 27 patients, the global Centiloid scale of amyloid deposition did not show a significant negative correlation with MMSE scores (*r* = −0.453, *p* = 0.221). In contrast, the global SUVR of tau deposition showed a significant negative correlation with MMSE scores (*r* = −0.554, *p* = 0.034), with the strongest correlation observed in the rest of the neocortex (*r* = −0.622, *p* = 0.007). On the other hand, the SUVR of tau deposition in the mesial temporal region did not show a significant correlation with MMSE scores (*r* = −0.406, *p* = 0.511) (Fig. 5; Table 2).

**Table 2:** Correlation between MMSE scores and Centiloid scale of amyloid-PET or SUVR of tau-PET with controlling age and sex

	Total 27 patients		14 patients with AD	
	Correlation coefficient	*p* value	Correlation coefficient	*p* value
Amyloid-PET Centiloid scale	−0.453	0.221	0.269	0.451
Tau-PET SUVR				
Global	−0.554	0.034	−0.609	0.038
Neocortex	−0.596	0.014	−0.689	0.014
Mesial temporal region	−0.406	0.511	0.158	0.763
Temporoparietal cortex	−0.546	0.041	−0.562	0.062
Rest of the neocortex	−0.622	0.007	−0.716	0.009

Among the 14 patients diagnosed with AD (A+T+), the global Centiloid scale of amyloid deposition did not show a significant negative correlation with MMSE scores (*r* = 0.269, *p* = 0.451). However, the global SUVR of tau deposition demonstrated a significant negative correlation with MMSE scores (*r* = −0.609, *p* = 0.038). A significant negative correlation was stronger between the neocortical tau deposition and the MMSE scores (*r* = −0.689, *p* = 0.014). The strongest correlation in this subgroup was observed in the rest of the neocortex (*r* = −0.716, *p* = 0.009). In contrast, the SUVR of tau deposition in the mesial temporal region did not show a significant correlation (*r* = 0.158, *p* = 0.763) (Fig. 5; Table 2).

## Discussion

Patients with cognitive impairment and clinically suspected AD were subjected to biomarker-based ATN classification using highly specific amyloid and tau PET ligands, along with MRI. As a result, 14 of the 27 patients tested positive for both amyloid and tau PET and were diagnosed with AD. This rate of AD diagnosis is consistent with previous studies utilizing amyloid and tau PET in memory clinics [16–18].

In this study, ^18^F-NAV4694 was used as the amyloid PET ligand. This ligand has a chemical structure similar to ^11^C-PiB and exhibits a high dynamic range with low nonspecific accumulation [19]. Therefore, it has been reported to be highly effective for detecting small amounts of amyloid deposition [20]. Additionally, the tau PET ligand ^18^F-MK6240 was used in this study. This ligand has been reported to bind specifically to 3R/4R tau and is unlikely to bind to 3R tau alone or 4R tau alone [21]. Furthermore, it does not accumulate in the choroid plexus, enabling the detection of tau deposition in the limbic system at lower Braak stages [22]. These properties make ^18^F-MK6240 ideal for diagnosing the T component in AD.

Amyloid and tau PET findings were concordant in 24 of 27 cases (89%). A significant correlation has been reported between amyloid PET and tau PET SUVR [23]. However, three cases showed discordance: one amyloid-positive, tau-negative patient, and two amyloid-negative, tau-positive patients. In the amyloid-positive, tau-negative case, the amyloid Centiloid scale was relatively low at 37.0, but significant atrophy was observed in the mesial temporal area, and the MMSE score was reduced to 22 points. This suggests the possible accumulation of pathological proteins other than 3R/4R tau (Fig. 3). The two amyloid-negative, tau-positive cases may represent primary age-related tauopathy with 3R/4R tau deposition and mesial temporal lobe atrophy, which mimics AD. Differentiating these cases from AD, where amyloid PET proves useful, is crucial (Fig. 4) [24].

In AD, many studies have reported that tau deposition is more closely associated with cognitive function than amyloid-β deposition [25–29]. Furthermore, the progression of Braak stages of tau deposition correlates with cognitive decline [27, 28]. This study observed no association between amyloid deposition and global cognitive function. In addition, when tau deposition was confined to the mesial temporal region at lower Braak stages, there was no association with cognitive function. On the other hand, when tau deposition extended to the neocortex, particularly the frontal lobes, the correlation with global cognitive function became stronger. This trend was more pronounced in the 14 cases diagnosed with AD compared to all 27 cases in the present study. These results align with the typical clinical phenotype of amnestic AD, where memory deficits dominate when tau is localized to the mesial temporal region. However, as tau accumulates in neocortical regions, additional symptoms such as language, navigation, and executive dysfunction emerge [8]. These findings suggest that while amyloid PET is critical for diagnosing AD, assessing neocortical tau deposition via tau PET is essential for predicting cognitive decline. This supports the hypothesis that anti-amyloid-β antibody therapy may be most effective at lower Braak stages. Conversely, if the disease has progressed with significant tau deposition in the neocortex, anti-amyloid-β therapy alone may have limited clinical efficacy [3].

This study has limitations. First, the small sample size restricts the ability to characterize individual ATN profiles. Second, there is a lack of pathological confirmation. The possibility of pathological protein deposition other than 3R/4R tau, which may not be detectable with ^18^F-MK6240, cannot be excluded. Third, this study may not have included patients with high cognitive reserve or complex pathology, potentially introducing patient selection bias. It has been reported that, in such patients, the degree of tau deposition does not necessarily correlate with cognitive function [30].

## Conclusion

Using highly specific amyloid and tau PET ligands, along with MRI, we were able to accurately classify ATN profiles in patients with suspected AD. Both amyloid-positive and tau-negative, as well as amyloid-negative and tau-positive cases, were identified, facilitating a more accurate diagnosis of AD. While amyloid deposition did not correlate with cognitive function, tau deposition—particularly in the neocortex—showed a significant correlation. Additionally, tau PET allowed the evaluation of tau deposition propagation in the neocortex beyond the mesial temporal region. These findings align with the revised criteria for AD diagnosis and staging which emphasize the location of tau deposition as detected by PET [30].

## Data Availability

The datasets used and/or analyzed during the current study are available from the corresponding author upon reasonable request.
